# Prevalence, Features and Risk Factors for Malaria Co-Infections amongst Visceral Leishmaniasis Patients from Amudat Hospital, Uganda

**DOI:** 10.1371/journal.pntd.0001617

**Published:** 2012-04-10

**Authors:** Erika van den Bogaart, Marieke M. Z. Berkhout, Emily R. Adams, Pètra F. Mens, Elizabeth Sentongo, Dawson B. Mbulamberi, Masja Straetemans, Henk D. F. H. Schallig, Francois Chappuis

**Affiliations:** 1 Department of Biomedical Research, Royal Tropical Institute (KIT), Amsterdam, The Netherlands; 2 Department of Medical Microbiology, Makerere University College of Health Sciences, Kampala, Uganda; 3 Ministry of Health, Kampala, Uganda; 4 Médecins Sans Frontières, Geneva, Switzerland; 5 Geneva University Hospitals and University of Geneva, Geneva, Switzerland; Emory University, United States of America

## Abstract

**Background and methodology:**

Due to geographic overlap of malaria and visceral leishmaniasis (VL), co-infections may exist but have been poorly investigated. To describe prevalence, features and risk factors for VL-malaria co-infections, a case-control analysis was conducted on data collected at Amudat Hospital, Uganda (2000–2006) by Médecins sans Frontières. Cases were identified as patients with laboratory-confirmed VL and malaria at hospital admission or during hospitalization; controls were VL patients with negative malaria smears. A logistic regression analysis was performed to study the association between patients' characteristics and the occurrence of the co-infection.

**Results:**

Of 2414 patients with confirmed VL, 450 (19%) were positively diagnosed with concomitant malaria. Most co-infected patients were males, residing in Kenya (69%). While young age was identified by multivariate analysis as a risk factor for concurrent VL and malaria, particularly the age groups 0–4 (odds ratio (OR): 2.44; 95% confidence interval (CI): 1.52–3.92) and 5–9 years (OR: 2.23, 95% CI: 1.45-3-45), mild (OR: 0.53; 95% CI: 0.32–0.88) and moderate (OR: 0.45; 95% CI: 0.27–0.77) anemia negatively correlated with the co-morbidity. VL patients harboring skin infections were nearly three times less likely to have the co-infection (OR: 0.35; 95% CI: 0.17–0.72), as highlighted by the multivariate model. Anorexia was slightly more frequent among co-infected patients (OR: 1.71; 95% CI: 0.96–3.03). The in-hospital case-fatality rate did not significantly differ between cases and controls, being 2.7% and 3.1% respectively (OR: 0.87; 95% CI: 0.46–1.63).

**Conclusions:**

Concurrent malaria represents a common condition among young VL patients living in the Pokot region of Kenya and Uganda. Although these co-morbidities did not result in a poorer prognosis, possibly due to early detection of malaria, a positive trend towards more severe symptoms was identified, indicating that routine screening of VL patients living in malaria endemic-areas and close monitoring of co-infected patients should be implemented.

## Author Summary

Visceral leishmaniasis (VL) and malaria are two major parasitic diseases sharing a similar demographic and geographical distribution. In areas where both diseases are endemic, such as Sudan, Uganda, India and Bangladesh, co-infection cases have been reported, but features and risk factors associated with these co-morbidities remain poorly characterized. In the present study, routinely collected data of VL patients admitted to Amudat Hospital, Uganda, were used to investigate the magnitude of VL-malaria co-infections and identify possible risk factors. Nearly 20% of the patients included in this study were found to be co-infected with VL and malaria, indicating that this is a common condition among VL patients living in malaria endemic areas. Young age (≤9 years) was identified as an important risk factor for contracting the VL-malaria co-infection, while being anemic or carrying a skin infection appeared to negatively correlate with the co-morbidity. Co-infected patients presented with slightly more severe symptoms compared to mono-infected patients, but had a similar prognosis, possibly due to early diagnosis of malaria as a result of systematic testing. In conclusion, these results emphasize the importance of performing malaria screening amongst VL patients living in malaria-endemic areas and suggest that close monitoring of co-infected patients should be implemented.

## Introduction

Due to extensive overlap in the geographical distribution of many infectious diseases, multiple infections appear to be the rule rather than the exception in many tropical and subtropical regions [1]. Polyparasitism, in particular, may predominate in rural areas of developing countries, where poor sanitation and economic conditions allow the uninterrupted transmission of many parasites [2]–[5]. Prevalences of multiparasite infections above 30% have been shown to occur regularly throughout South-East Asia and much of Central and West Africa, with communities harboring multiple parasites in up to 80% of their population [6]. Importantly, the different combinations in which pathogens might co-exist in a certain population and their distribution therein, do not result from a random process, but they are rather part of a selection governed by a variety of ecological and host factors, which include the biological interactions of the parasites within the host [3], [7]. These interactions may affect the pathogenicity of the infective agents, resulting in a spectrum of effects on the polyparasitized host, ranging from exacerbation to amelioration of disease severity [8]. Despite the recent upsurge in investigations targeting multiple helminth species and *Plasmodium*-helminth co-infections [9]–[12], the actual extent of polyparasitism and its pathological consequences remain largely unassessed [8]. The scarcity of information is particularly striking for infections that are clinically not apparent or lack pathognomonic signs. As the tropics and sub-tropics are burdened with infectious diseases sharing similar clinical pictures, recognition of these diseases occurring in the same patient might be difficult in poor resource settings.

Visceral leishmaniasis (VL) is a life-threatening syndrome caused by protozoan parasites of the *Leishmania donovani* complex. Most cases occur in East Africa, South-East Asia and Brazil, where nearly 0.5 million people get infected each year [13], half of whom are children [14]. Differential diagnosis of VL often includes malaria amongst other febrile splenomegalies, due to its geographical and clinical overlap. Malaria, in fact, is widespread in tropical and sub-tropical regions of the world, where it accounts for more than 250 million cases annually, the vast majority of which occurs among children under 5 years old [15]. Transmission can occur throughout the year or be seasonal, depending on the region [16]. In the latter case, transmission seasons for VL and malaria may not coincide, but the two diseases still overlap, due to the longer incubation period of VL. The overlap in disease distribution suggests the two diseases could co-occur in the same host. Nonetheless, figures describing the extent of VL and malaria co-infections are not readily available in literature.

To gather evidence on the occurrence of such co-morbidities, a systematic review of the present literature was first conducted (Figure S1). This review showed that cases of VL and malaria co-infections have been reported across various African and Asian countries, with the prevalence among VL patients ranging from 20.8% and 6.4% in Uganda [17], [18] to 10.7% in Sudan [19] and 1.2% in Bangladesh [20] and a rate of 5.9% among Indian patients with fever and splenomegaly [21]. With the exception of the case-reports [22]–[25], whose evidence remains anecdotal, no further details on these co-infections are described, preventing identification of possible risk factors and specific features associated with these co-morbidities. To address this issue, a retrospective case-control analysis was performed on clinical data of VL patients living in the Pokot territory of Kenya and Uganda and admitted to Amudat Hospital, North-Eastern Uganda. The area, a semi-arid lowland region mainly inhabited by pastoralists of the Pokot tribe, is part of a large VL endemic focus, which includes Pokot County in Uganda and extends eastwards to Pokot North, West Pokot, East Pokot and Baringo Districts in Kenya [26]. Here, VL is caused by *Leishmania donovani* and transmitted by the sandfly *Phlebotomus martini* [26]. Malaria represents another major health problem in the region, with incidence rates ranging from 20% in Kenya to 30% in Uganda [15]. Hookworms and other neglected tropical diseases such as lymphatic filariasis have also been identified as endemic in the area [27]. Given such high disease burdens, it is hardly surprising that polyparasitism may represent a common condition.

The main goal of the present study was to describe the prevalence of VL and malaria co-infections amongst VL patients attending Amudat hospital and identify risk factors associated with this condition. Recognition of the burden posed by these co-morbidities among different patient groups may contribute to improve the clinical management of VL in malaria-endemic areas.

## Methods

### Ethics statement

The analysis was conducted on anonymized data, collected as part of routine patient care; no additional investigations were performed. Therefore, no prior informed consent from the patients was required. Ethical approval for the study was obtained from the MSF Ethics Review Board (8^th^ April 2011).

### Patients

Data of suspected VL patients admitted to Amudat Hospital, a 120-bed rural hospital located in Pokot County, Amudat District, Uganda, were collected between January 2000 and December 2006 by Médecins sans Frontières (MSF-Swiss Section). MSF support to Amudat Hospital medical activities included the establishment of a VL control program, in which patients were provided with free diagnosis and treatment. Clinically suspected VL patients, defined as individuals with a history of prolonged fever (≥14 days) associated with either splenomegaly or wasting, were included in MSF's program and further examined for VL. According to the diagnostic algorithm implemented [28], VL was confirmed either serologically and/or parasitologically. Serological tests included the direct agglutination test (DAT) and after 2004, the rk39 antigen-based DiaMed IT-Leish dipstick, while parasitological confirmation was obtained by microscopy examination of spleen or, more rarely, lymph node aspirates. Microscopic examination of thick and thin blood smears for malaria detection was systematically performed at hospital admission or during hospitalization. The diagnosis of other concomitant diseases was based on clinical suspicion, possibly supported by laboratory-confirmation. Testing for HIV was performed only occasionally, due to the lack of voluntary counseling and testing facilities and the shortage of antiretroviral treatment in the district. First-line treatment for primary VL consisted of intramuscular meglumine antimoniate (Glucantime™), temporarily replaced, due to drug shortages, by intravenous ampothericin B deoxycholate or intramuscular sodium stibogluconate (Pentostam™). Second-line treatment based on amphotericin B deoxicholate was administered in case of relapse or intolerance to antimonials. Chloroquine in combination with sulfadoxine-pyrimethamine or alternatively quinine was administered as first-line treatment for malaria. Demographic and medical data of all VL suspected patients were collected by MSF and the local medical staff and entered in a Microsoft Excel data sheet. Besides outcomes of the performed laboratory tests, the database gathered information on patients' medical history, clinical presentation on admission, symptoms, clinically suspected co-morbidities, treatment administered and relative outcome.

### Data analysis

A retrospective case-control analysis was performed. Cases were identified as patients with a laboratory-confirmed diagnosis of both VL and malaria at hospital admission or during hospitalization; controls were patients similarly diagnosed with VL, whose blood smears tested negative for malaria. Descriptive analyses were conducted to feature the overall study population and the co-infected patients and to assess prevalence and mortality rates of the co-infection. Statistical analysis was performed using SPSS 15.0 software (SPSS Inc., Chicago, IL, USA). Univariate analyses exploring the association between the explanatory variables and the outcome were performed, using the Pearson Chi-square test. Specifically, risk factors, symptoms and the in-hospital death were examined for their association with the co-infection. Associations are shown as odds ratios (OR) with 95% confidence intervals (CI). Denominators can vary for each variable because of incomplete data in the database. Continuous variables were categorized into predefined groups: age (0–4, 5–9, 10–19, 20–29, ≥30 years) based on malaria risk, anemia on hemoglobin level (severe <5.3 g/dl, moderate 5.3–7.2 g/dl, mild 7.3–10.9 g/dl, none ≥11 g/dl) and spleen size beneath the costal margin by tertiles (<11 cm, 11–14 cm, >14 cm). Seasonality of the co-infection was examined by categorizing hospital admissions during wet season (from March to May, from October to November) and dry season (from December to February, from June to September), which reflects the climate in Uganda. Anthropometric indices –WHZ, weight-for-height Z-score for children ≤5 years, BMIZ, body mass index Z-score for children 6–19 years and BMI for patients >19 years– were used to categorize nutritional status, according to the WHO standards (severe malnutrition: WHZ and BMIZ<−3SD, BMI<16 kg/m^2^; moderate-mild malnutrition: WHZ and BMIZ<−2SD, BMI 16.0–18.4 kg/m^2^; no malnutrition: WHZ and BMIZ>−2SD, BMI≥18.5 kg/m^2^) [29]. Self-reported symptoms experienced prior to hospitalization were included in the analysis, with the exception of the variable diarrhea, which combined episodes that occurred prior to and during hospitalization. In order to identify independent patient characteristics associated with the co-infection, logistic regression models were constructed, using a manual stepwise backward procedure to control for confounding and describe statistical interactions. All variables with a *P*-value<0.10 in the univariate analyses were entered stepwise in the multivariate models. If an additional variable significantly increased the model fit (as assessed by the −2 Log Likelihood test) the variable was retained in the multivariate analysis (P<0.05).

## Results

### Prevalence, features and mortality rate of VL- malaria co-infections

Between 2000 and 2006, a total of 4428 VL suspected patients were admitted to Amudat Hospital, 57% of whom (*n* = 2511) were confirmed with VL. The diagnosis was confirmed by DAT in 1160 patients (46.2%), by DiaMed IT-Leish in 1115 patients (44.4%) and by parasitological evidence in a spleen or lymph node aspirate in 236 patients (9.4%). Of the 2511 VL-confirmed cases, 2461 (98%) were primary-VL infections, while the remaining 50 were relapsing-cases. 547 (22%) of the VL-confirmed patients were co-diagnosed with malaria at hospital admission or during hospitalization. Clinically-based diagnosis with negative or no microscopy confirmation occurred for 97 patients, who were excluded from the analysis. Only microscopy-confirmed cases of concomitant malaria were retained for analysis in the co-infected group, resulting in 19% of VL-confirmed patients (*n* = 450 out of 2414) co-infected with positively diagnosed malaria. Among the non-VL cases, 1107 patients (25%) were clinically diagnosed with probable hyper-reactive malarial splenomegaly, a severe syndrome frequently observed among patients exposed to persistent malarial antigenic stimulation. Most co-infected patients were males residing in Kenya (69%), with a median age of 10 years (inter quartile range 6–16) (table 2). With the highest percentage of cases found among children aged between 0 and 4 years (26%, *n* = 82/316) and a progressive decrease with age (13%, *n* = 32/251 in the age group ≥30 years) (table 1), the odds of detecting malaria in patients already diagnosed with VL appeared to be inversely related to age.

**Table 1:** Prevalence of VL-malaria co-infections, stratified by age, amongst VL patients from Amudat Hospital, Uganda, 2000–2006.

	Total (n = 2392)*	
Age group	%	*n* cases/ denominator
0–4 years	25.9	82/316
5–9 years	22.8	133/584
10–19 years	17.4	145/832
20–29 years	13.4	55/409
≥30 years	12.7	32/251

*: 22 missing values for the variable age.

**Table 2:** Characteristics associated with visceral leishmaniasis-malaria co-infections at Amudat Hospital, Uganda, 2000–2006 (part I).

Variable	Malarial-VL co-infection cases		Non-malarial VL cases		Crude Odds Ratio	95% Confidence Interval
	*n* (%)		*n* (%)			
**Gender**	**450**		**1964**			
Male	311	(69.1)	1350	(68.7)	1	
Female	139	(30.9)	614	(31.3)	0.98	0.79–1.23
**Age**	**447**		**1945**			
Median (inter-quartile range)	10 (6–16)		12 (7–21)			
0–4 years	82	(18.3)	234	(12.0)	2.34	1.53–3.75*
5–9 years	133	(29.8)	451	(23.2)	2.02	1.33–3.07*
10–19 years	145	(32.4)	687	(35.3)	1.44	0.96–2.18*
20–29 years	55	(12.3)	354	(18.2)	1.06	0.67–1.70
≥30 years	32	(7.2)	219	(11.3)	1	
**Country of origin**	**450**		**1962**			
Uganda	140	(31.1)	533	(27.2)	1	
Kenya	310	(68.9)	1429	(72.8)	1.21	0.97–1.51
**Season on admission**	**450**		**1964**			
Wet season	177	(39.3)	828	(42.2)	1	
Dry season	273	(60.7)	1136	(57.8)	1.12	0.91–1.39
**VL infection**	**450**		**1964**			
Primary infection	442	(98.2)	1922	(97.9)	1	
Relapse	8	(1.8)	42	(2.1)	0.83	0.39–1.78
**Previous VL treatment**	**450**		**1964**			
No	440	(97.8)	1928	(97.7)	1	
Yes	10	(2.2)	46	(2.3)	0.95	0.45–1.89
**Malnutrition**	**424**		**1882**			
No	168	(39.6)	638	(33.9)	1	
Moderate-mild	142	(33.5)	744	(39.5)	0.73	0.57–0.93*
Severe	114	(26.9)	500	(26.6)	0.87	0.66–1.13
**Anemia degree on admission**	**447**		**1938**			
Median hemoglobin g/dl (inter-quartile range)	7.3 (6.7–9.7)		7.3 (6.7–8.7)			
None (Hemoglobin ≥11 g/dl)	26	(5.8)	68	(3.5)	1	
Mild (Hemoglobin 7.3–10.9 g/dl)	276	(61.7)	1218	(62.8)	0.59	0.37–0.95*
Moderate (Hemoglobin 5.3–7.2 g/dl)	130	(29.1)	592	(30.5)	0.64	0.35–0.94*
Severe (Hemoglobin<5.3 g/dl)	15	(3.4)	60	(3.1)	0.91	0.32–1.35*
**Spleen size on admission**	**443**		**1943**			
Median spleen size below the costal margin (inter-quartile range)	13.0 (10.0–15.0)		13.0 (10.0–16.0)			
<11 cm	111	(25.1)	513	(26.4)	1	
11–14 cm	191	(43.1)	752	(38.7)	1.17	0.91–1.52
>14 cm	141	(31.8)	678	(34.9)	0.96	0.73–1.26

*: *P*-value<0.10 based on Person Chi-square tests.

Nearly all co- and mono-infected patients were diagnosed with a degree of anemia (hemoglobin level <11 g/dl) on hospital admission, with a median hemoglobin level of 7.3 g/dl (inter quartile range 6.7–9.7 for cases and 6.7–8.7 for controls) (table 2). Splenomegaly, as detected here by palpation of the spleen below the left costal margin, was also a common feature among co-infected patients, but neither its frequency (75% for cases, 74% for controls) nor its severity (median spleen size below the costal margin 13.0 cm for cases and controls) were increased by the malaria co-infection (table 2). More than half of the co-infected patients were co-diagnosed with a third infectious disease, reflecting the high prevalence of infections found in the study area. Acute respiratory infections followed by otorhinolaryngological and skin infections were the most common co-morbidities among both cases and controls (table 3).

**Table 3:** Characteristics associated with visceral leishmaniasis-malaria co-infections at Amudat Hospital, Uganda, 2000–2006 (part II).

Variable	Malarial-VL co-infection cases		Non-malarial VL cases		Crude Odds Ratio	95% Confidence Interval
	*n* (%)		*n* (%)			
**Concomitant diagnoses**	**450**		**1964**			
Acute respiratory infections	165	(36.7)	653	(33.2)	1.09	0.94–1.44
Ear, nose and throat infections#	46	(10.2)	234	(11.9)	1.01	0.73–1.26
Skin infections†	9	(2.0)	87	(4.4)	0.44	0.22–0.88*
Bacterial infections‡	7	(1.6)	15	(0.8)	1.91	0.83–5.07
Chicken pox	3	(0.7)	19	(1.0)	2.04	0.20–2.33
Tuberculosis	2	(0.4)	7	(0.4)	0.75	0.26–6.03
Brucellosis	3	(0.5)	15	(0.8)	0.71	0.21–2.46
Typhoid fever	0		9	(0.5)	1.00	0.99–1.00
Intestinal parasites§	2	(0.4)	34	(1.7)	0.25	0.06–1.06*
Hepatopathy	1	(0.2)	8	(0.4)	0.55	0.07–4.37
Other infections	4	(0.9)	37	(1.9)	0.47	0.17–1.32
**Symptoms on admission**	**81**		**908**			
Cough	34	(42.0)	380	(41.9)	1.01	0.63–1.59
Weight loss	29	(35.8)	267	(29.4)	1.34	0.83–2.16
Anorexia	19	(23.5)	129	(14.2)	1.85	1.07–3.20*
Bleeding	15	(18.5)	192	(21.1)	0.85	0.47–1.52
Headache	13	(16.0)	164	(18.1)	0.87	0.47–1.61
Malaise	14	(17.3)	100	(11.0)	1.69	0.92–3.11*
Diarrhea	23	(5.1)	165	(8.4)	0.59	0.38–0.92*
Vomiting	3	(3.7)	12	(1.3)	2.87	0.79–10.39*
Fatigue	2	(2.5)	25	(2.8)	0.89	0.21–3.85
Abdominal distension	1	(1.2)	11	(1.2)	1.02	0.13–8.00
Edema	1	(1.2)	26	(2.9)	0.42	0.06–3.17
Abdominal pain	1	(1.2)	14	(1.5)	0.80	0.10–6.15
Jaundice	1	(1.2)	10	(1.1)	1.12	0.14–8.88
Joint pain	0		12	(1.3)	1.00	0.99–1.00
Sweating	1	(1.2)	8	(0.9)	1.41	0.17–11.39
Palpitations	0		3	(0.3)	1.00	0.99–1.00
Skin rash	0		3	(0.3)	1.00	0.99–1.00

*: *P*-value<0.10 based on Person Chi-square tests.

# Ear, nose and throat infections include: otitis, pharyngitis, sinusitis, tonsillitis, parotitis, gingivitis and noma.

**†:** Skin infections include bacterial and fungal infections.

**‡:** Bacterial infections include: infections of the urinary tract, sepsis and other infections.

**§:** Intestinal parasite infections include protozoan and helminthic infections.

Of the 450 co-infected patients, 2.7% (*n* = 12) died while hospitalized. A similar in-hospital mortality rate was found in the control group (3.1%, *n* = 60) (OR: 0.87; 95% CI: 0.46–1.63) (data not shown).

### Risk factors for VL-malaria co-infections

Associations between demographic and clinical variables and the co-infection, as described by the univariate analyses, are summarized in Tables 2 and 3. Young age (≤9 years) was identified as a risk factor for the VL-malaria co-infection: in particular, children in the age groups 0–4 (OR: 2.34; 95% CI: 1.53–3.75) and 5–9 years (OR: 2.02; 95% CI: 1.33–3.07) were more than two times more likely to be co-infected compared to adults ≥30 years. Transmission seasons for malaria and VL did not coincide in the study area. However, no significant difference was found in the seasonal distribution of cases' and controls' hospitalizations. VL-relapsing patients did not show different susceptibilities to the malarial co-morbidity, compared to patients with primary-VL infections, and were therefore maintained in the relative groups (cases and controls). Malnourishment was relatively more common in the control group, resulting in patients with mild-moderate malnutrition being negatively associated with the co-infection, compared to the well-nourished patients (OR: 0.73; 95% CI: 0.57–0.93). A significant negative association was found between anemia and the co-infection: patients whose hemoglobin level was <11 g/dl, in fact, were less likely to be diagnosed with the co-infection compared to the normochromic group. Among concomitant diseases, patients diagnosed with a skin infection (OR: 0.44, 95% CI: 0.22–0.88) were less likely to have the VL-malaria co-infection. A trend towards a negative association was also observed for patients suspected to harbor intestinal protozoa and/or helminthes, possibly indicating a mutual protection among the three diseases (OR: 0.25, 95% CI: 0.06–1.06). Finally, anorexia (OR: 1.85, 95% CI: 1.07–3.20), malaise (OR: 1.69, 95% CI: 0.92–3.11), and vomiting (OR: 2.87, 95% CI: 0.79–10.39) were identified as positively associated symptoms of the VL-malaria co-infection, while a negative association was found for diarrhea (OR: 0.59, 95% CI: 0.38–0.92).

All 9 variables associated with the co-infection in the univariate analyses were included in the multivariate logistic regression models. Two separate models were created, due to the high number of missing data in reporting the symptoms: model A, based on 2306 patients, which includes all variables except the symptoms and model B, based on 929 patients, which combines symptoms and all other variables. Model A (table 4) shows that, after adjusting for the other variables in the model, mild-moderate malnutrition lost its statistical significance (*P*-value 0.18). However, the variable was kept in the final model as it was found to be a confounder for age. After adjusting for the other variables included in the model, age ≤9 years remained significantly associated with the co-infection. When compared to adults ≥30 years, the risk of being diagnosed with the co-morbidity was highest among children aged 0–4 years (OR: 2.44; 95% CI: 1.52–3.92) and progressively decreased with age (OR: 2.23; 95% CI: 1.45–3.45 for age group 5–9 years; OR: 1.48; 95% CI: 0.97–2.26 for age group 10–19 years). Moderate (OR: 0.45; 95% CI: 0.27–0.77) and mild anemia (OR: 0.53; 95% CI: 0.32–0.88) negatively correlated with the co-infection. Finally, the model highlighted a negative association between being co-infected with VL and malaria and carrying a skin infection (OR: 0.35; 95% CI: 0.17–0.72) and, to a lesser extent, intestinal parasites (OR: 0.28; 95% CI 0.07–1.19). Patients suspected to harbor such pathogens, in fact, were three times less likely to be diagnosed with the co-infection.

**Table 4:** Model A: multivariate analysis of risk factors for VL-malaria co-infections among 2306 Amudat Hospital patients.

Variable	Odds Ratio	95% Confidence Interval	*P*-value
**Age**			
0–4 years	2.44	1.52–3.92	<0.010
5–9 years	2.23	1.45-3-45	<0.010
10–19 years	1.48	0.97–2.26	0.067
20–29 years	1.09	0.67–1.76	0.730
≥30 years	1		<0.001
**Malnutrition**			
No	1		0.295
Moderate-mild	0.84	0.64–1.08	0.176
Severe	1.01	0.77–1.33	0.955
**Anemia on admission**			
None (Hb≥11 g/dl)	1		0.035
Mild (Hb 7.3–10.9 g/dl)	0.53	0.32–0.88	0.014
Moderate (Hb 5.3–7.2 g/dl)	0.45	0.27–0.77	0.004
Severe (Hb<5.3 g/dl)	0.49	0.22–1.08	0.075
**Concomitant diagnoses**			
Skin infections	0.35	0.17–0.72	0.005
Intestinal parasites	0.28	0.07–1.19	0.085

In model B (table 5), logistic regression was performed on the sub cohort of patients for whom symptoms, besides the other variables, were reported. As anemia was found to be a confounder in the association between age and the co-infection, the variable was kept in the model. Besides concomitant diagnoses, diarrhea, vomiting and malaise lost their significance and were consequently excluded from the model. The association between anorexia and the co-infection also lost its statistical significance in the multivariate analysis, but the variable was kept in the model as it increased the model fit. A positive association with this symptom can be seen (OR: 1.71; 95% CI: 0.96–3.03), indicating that co-infected patients more frequently reported anorexia among their symptoms.

**Table 5:** Model B: multivariate analysis of symptoms associated to VL-malaria co-infections among 929 Amudat Hospital patients.

Variable	Odds Ratio	95% Confidence Interval	*P*-value
**Age**			
0–4 years	3.19	1.09–9.32	0.034
5–9 years	2.99	1.10–8.13	0.032
10–19 years	0.86	0.31–2.45	0.783
20–29 years	0.85	0.26–2.77	0.785
≥30 years	1		<0.001
**Malnutrition**			
No	1		0.522
Moderate-mild	1.10	0.61–1.98	0.748
Severe	1.41	0.77–2.60	0.265
**Anemia on admission**			
None (Hb≥11 g/dl)	1		0.199
Mild (Hb 7.3–10.9 g/dl)	0.31	0.06–1.54	0.154
Moderate (Hb 5.3–7.2 g/dl)	0.24	0.05–1.21	0.083
Severe (Hb<5.3 g/dl)	0.19	0.03–1.38	0.101
**Symptoms**			
Malaise	2.14	1.12–4.07	0.021
Anorexia	1.71	0.96–3.03	0.070

## Discussion

Nineteen percent of the VL-confirmed patients admitted to Amudat Hospital between 2000 and 2006 were co-diagnosed with malaria. Young age (≤9 years) was identified as a risk factor for concomitant VL and malaria, while being anemic or having a skin infection appeared to negatively correlate with the co-infection. Anorexia was slightly more frequent among co-infected patients compared to the mono-infected ones. The in-hospital case-fatality rate did not significantly differ between cases and controls, indicating that VL-malaria co-infections did not result in a poorer prognosis.

Affecting nearly one out of five VL patients, VL and malaria co-infections appeared to be a common condition among VL patients living in the Pokot Region of Kenya and Uganda. Though in agreement with the findings of Mueller *et al.* who investigated the in-hospital mortality of VL within the same study population [17], the co-infection rate described in the present population (19%) is one of the highest so far reported, as identified by our systematic literature review. The main reason behind this variation may be found in the malaria prevalence characterizing the study areas (31% and 18% in Uganda and Kenya, respectively, 28% in Sudan, 0.31% in India and 2.0% in Bangladesh in the corresponding study years [15], [30]). This case, however, does not apply to the study of Kolaczinski *et al.* [18], in which only 6.4% of the Amudat Hospital VL patients recruited between June and September 2006 were reported to be co-infected with malaria. Although different malaria diagnostics were used to define the two study populations (microscopy in the present study and the *Plasmodium falciparum*-specific Paracheck test in Kolaczinski's study), the role played by the use of such diagnostic tools is likely to be marginal, considering the high performance of the Paracheck test in Uganda [31], where *P. falciparum* causes most of malaria infections [15]. Most likely, the higher co-infection prevalence observed here resulted from recruiting a larger number of study subjects. When examining only patients hospitalized in the same four-month period of 2006, indeed, the co-infection rate remains significantly higher (19%), even in comparison with the malaria prevalence estimated by Kolaczinski *et al.* (16%), emphasizing the importance of recruiting large numbers of participants.

The co-infected population described by the present study mainly consisted of male Kenyans (69%), with a median age of 10 years. Despite the similar demographic pattern found among the controls, young age appeared to be positively associated with the co-infection; children ≤9 years, in fact, were found to be more than two times more likely to be co-infected with malaria, compared to adults ≥30 years, reflecting the well-known age patterns of malaria [32].

Anemia, a hallmark of both VL and malaria, negatively correlated with the co-occurrence of malaria in VL patients. Given the bi-directional nature of such association, it is not possible to determine *a priori* whether such correlation might reflect the reduced susceptibility of VL anemic patients to the malarial infection or simply result from the interaction of the two diseases upon each another. Splenic-associated hemolysis and reduced hematopoiesis, as a result of abnormal iron retention by macrophages, have been implicated in the multifactorial origin of VL-induced anemia, where both hemoglobin and plasma iron levels are affected [33]. Iron-deficiency, in particular, was associated with delay in the *in vitro* intraerythrocytic growth of *Plasmodium* parasites [34] and protection from mild clinical malaria *in vivo* [35]. Although in our study population, anemia was diagnosed on the basis of the hemoglobin levels only, iron deficiency might be equally common, due to poor nutritional status and ongoing diseases, providing temporary resistance to the malaria disease. A less severe course of VL as a result of concomitant malaria might represent a possible scenario, corroborated by the findings that severe splenomegaly and fatal outcome were slightly less common among co-infected patients. The milder anemia observed among co-infected patients compared to mono-infected patients could then be the result of a malaria-mitigating effect on VL. Such hypothesis, however, appears to be in contrast with previous observations obtained from animal models, in which the course of *Leishmania* infections appeared to be unaffected or exacerbated by the concomitant presence of *Plasmodium* parasites [36]–[39]. In addition, the more frequent reporting of anorexia by co-infected patients seems to point towards an exacerbation of disease severity rather than alleviation. The hypothesis whereby co-infected patients may be diagnosed at earlier stage than VL mono-infected patients, due to more severe symptoms, is unlikely, as no difference was found in the ongoing sickness duration of cases and controls prior to hospitalization. The severe dehydration observed in some patients may have led to misleading (elevated) hemoglobin levels on hospital admission. However, the significant increase in hemoglobin level observed after treatment (+2.14 g/dl) suggests that severe dehydration may have played only a marginal role. Finally, it should be noted that the groups comprising the non-anemic patients consisted of relatively few patients for both cases and controls, which may limit the validity of the associations described. In fact, when patients with no anemia and mild anemia (the most numerous group) are grouped together, the association between moderate anemia and the VL-malaria co-infection looses its significance (data not shown).

Unlike what may be observed in other co-morbidities [40], [41], co-infections with VL and malaria did not result in an exacerbated spleen enlargement compared to VL mono-infections. This may be related to splenic disorders promoted by VL, which might be hampering for the malaria-induced hyperplasia. Remarkably, while massive splenomegaly is a common feature of VL, it is more rarely associated to malarial infections, which instead induce only moderate spleen enlargement. Therefore, exacerbation of splenomegaly as promoted by concomitant malaria might have occurred but be undetectable due to the larger effect produced by VL on the spleen size. Finally, a possible malaria-induced attenuation of VL severity should not be excluded.

More than half of the patients among both cases and controls were co-diagnosed with one or more infections beyond VL and malaria. If this figure reflects the high infectious disease burdens found in the study area, we cannot exclude the development of co-morbidities to be related to the VL infection. Importantly, if VL might have favored the superimposing of other infections, malaria neither exacerbated the susceptibility of co-infected patients nor influenced the concomitant disease-pattern, with the exception of skin infections and, to a lesser extent, intestinal parasites. VL patients diagnosed with bacterial and fungal skin infections, in fact, appeared to be more than two times less likely to be co-diagnosed with malaria, although no evident explanation could be found. Similarly, a negative, but not significant association between co-infected patients and VL patients suspected to harbor intestinal parasites was identified, suggesting that the parasites involved (intestinal protozoa and/or helminthes, *Leishmania* and *Plasmodium* spp) may be able to cross-regulate their host susceptibility. It should be noted, however, that diagnosis of intestinal parasitic infections for some patients might have been based on clinical suspicion only, with no laboratory-confirmation, and that testing for these parasites might have not been performed systematically, as suggested by the very low prevalence of positive patients (1.5%). A dedicated study recruiting larger patient cohorts diagnosed with intestinal parasitic infections and performing systematic testing of all study subject would be required to confirm this finding.

Anorexia was reported slightly more frequently in the co-infected group than in the mono-infected group, indicating that a more severe clinical picture may be associated with the overlapping of the two diseases. This symptom was frequently associated with the malarial infection; however, the significance of such finding remains questionable, given the relatively small number of patients which it is based on. Importantly, if VL-malaria co-infections were associated with a worse symptomatology, they did not result in a poorer prognosis among patients properly diagnosed and treated. The overall fatality-rate, in fact, was similar in the co-infected group as in the mono-infected one, suggesting that here, malaria and its treatment neither altered the severity of VL and its responsiveness to chemotherapy nor resulted in an increased toxicity of the anti-leishmanial drugs. A similar conclusion might be extended to the co-infected patients diagnosed with relapses rather than primary infections, as no death occurred among these patients. Whether the prognosis of co-infected patients in whom malaria remained undetected and/or untreated may be different, is hard to predict, but a higher mortality risk may be expected, as observed for late diagnosed malaria.

The findings highlighted by the present study may be generalized with caution to the VL-symptomatic population residing in the Uganda-Kenya VL foci. Evidence in support of this statement includes the large catchment area of Amudat Hospital, its specialization in VL and the local awareness of the disease. Until November 2006, when MSF transferred its medical activities to Kacheliba Health Centre in Kenya, Amudat Hospital was the only reliable facility available in the region for the diagnosis and treatment of VL. Furthermore, a recent study conducted in the same area indicated that VL is a well-known disease among members of the Pokot communities, who not only recognize its main clinical signs and poor prognosis, but are also aware that drug treatment is available and effective [18]. This resulted in many individuals with symptoms of VL presenting voluntarily to the MSF's VL-ward, where VL diagnosis and treatment were provided free of charge by MSF.

The main limitations of this study relate to data quality and lack of mono-infected malaria patients and healthy controls. It was not possible to assess the malaria prevalence in the study area, which prevented us from calculating the VL associated risk of being-co-diagnosed with malaria. Comparison of study variables was only possible for VL co-and mono-infected patients, possibly reducing the significance of some of the associations found. The lack of quality control on malaria microscopy might have led to poor performance, possibly affecting the accuracy of the study results. Hemoglobin estimates were only semi-quantitative and possibly subject to interpretation, been based on the Lovibond method. Spleen size measurement might have suffered from poor standardization, with different physicians using slightly different techniques.

In conclusion, this study highlights for the first time that concurrent VL and malaria infection represents a common co-morbidity among young patients living in highly malaria-endemic areas, such as the Pokot territory of Kenya and Uganda. Based on these findings, we recommend that malaria screening be implemented for all VL patients residing in malaria-endemic areas in order to promptly initiate antimalarial drug treatment. Future studies are needed to address the complicated question on whether the concurrency of such infections might influence the course of one or both diseases in humans. This may be of importance for the design of successful disease control programs.

## Supporting Information

Figure S1 **Search strategy and study selection used for the systematic literature review.**
(DOC)
