# Supplementary material for: Prevalence, Features and Risk Factors for Malaria Co-Infections amongst Visceral Leishmaniasis Patients from Amudat Hospital, Uganda
Source: PLoS Negl Trop Dis. 2012 Apr 10;6(4):e1617. doi: 10.1371/journal.pntd.0001617 (PMC3323524; doi:10.1371/journal.pntd.0001617)
Supplement: Figure S1 — Search strategy and study selection used for the systematic literature review. (DOC) [file pntd.0001617.s001.doc]

474 studies identified by searching Medline/Pubmed (1950-week 19, 2011), Embase (1947-week 19, 2011), Google Scholar, African Journals Online and African Index Medicus -Access to African Health Information- electronic databases. A combination of search terms tailored to individual database was used, with no restriction on study design or publication language. Search terms included: *Leishmania*, leishmaniasis, VL, malaria, *Plasmodium*, prevalence, incidence, risk factors, morbidity and mortality.

407 studies screened for their eligibility (titles and abstracts or full-texts) after removal of duplicates.

Criteria for eligibility included:

concomitant exposure to VL and malaria

exposure of human subjects

399 studies excluded

10 full-text articles assessed for eligibility

3 studies excluded.

Reasons for exclusion:

- Full-text cannot be retrieved: 2

- Lack of relevance: 1

7 studies included in the systematic review

5 additional studies identified through manual screening of conference proceedings and references of retrieved articles

5 full-text articles assessed for eligibility

2 studies included in the systematic review

3 studies excluded.

Reasons for exclusion:

- Full-text cannot be retrieved: 2

- Lack of relevance: 1

9 studies included in the systematic review: 5 cross-sectional studies and 4 case-report [16-24]

Figure S1: Search strategy and study selection used for the systematic literature review.
